# A novel inflammatory biomarker, high-sensitivity C-reactive protein-to-albumin ratio, is associated with 5-year outcomes in patients with type 2 diabetes who undergo percutaneous coronary intervention

**DOI:** 10.1186/s13098-022-00977-9

**Published:** 2023-02-06

**Authors:** Jiawen Li, Pei Zhu, Yulong Li, Kailun Yan, Xiaofang Tang, Jingjing Xu, Weixian Yang, Shubin Qiao, Yuejin Yang, Runlin Gao, Bo Xu, Jinqing Yuan, Xueyan Zhao

**Affiliations:** National Clinical Research Center for Cardiovascular Diseases and State Key Laboratory of Cardiovascular Diseases, Fu Wai Hospital, National Center for Cardiovascular Diseases, Chinese Academy of Medical Sciences and Peking Union Medical College, No. 167 Beilishi Road, Xicheng District, Beijing, 100037 China

**Keywords:** hs-CRP-to-albumin ratio, Type 2 diabetes mellitus, Percutaneous coronary intervention, Dual antiplatelet therapy

## Abstract

**Background:**

Patients with coronary artery disease (CAD) combined with diabetes have a higher risk of cardiovascular events, and high-sensitivity C-reactive protein (hs-CRP)-to-albumin ratio (CAR) is a novel inflammatory biomarker. However, whether the CAR can identify high-risk patients with CAD and type 2 diabetes (T2DM) remains unclear.

**Methods:**

The present study was based on a prospective and observational cohort with 10,724 individuals who undergo percutaneous coronary intervention (PCI) in Fu Wai Hospital throughout the year 2013 consecutively enrolled. The primary endpoint was all-cause mortality. The secondary endpoint was cardiac mortality. CAR was calculated with the formula: hs-CRP (mg/L)/albumin (g/L). According to the optimal cut-off value of CAR for all-cause mortality, patients were divided into higher CAR (CAR-H) and lower CAR (CAR-L) groups.

**Results:**

A total of 2755 patients with T2DM who underwent PCI and received dual antiplatelet therapy were finally enrolled. During a follow-up of 5 years (interquartile range: 5.0–5.1 years), 126 (4.6%) all-cause mortalities and 74 (2.7%) cardiac mortalities were recorded. In the multivariable Cox model, CAR-H was associated with a higher risk of all-cause mortality (hazard ratio [HR]: 1.634, 95% confidence interval [CI] 1.121–2.380, p = 0.011) and cardiac mortality (HR: 1.733, 95% CI 1.059–2.835, p = 0.029) compared with CAR-L. When comparing the predictive value, CAR was superior to hs-CRP for all-cause mortality (area under the curve [AUC] 0.588 vs. 0.580, p = 0.002) and cardiac mortality (AUC 0.602 vs. 0.593, p = 0.004).

**Conclusion:**

In this real-world cohort study, a higher level of CAR was associated with worse 5-year outcomes among diabetic patients with PCI.

**Supplementary Information:**

The online version contains supplementary material available at 10.1186/s13098-022-00977-9.

## Introduction

Globally, approximately 32.2% of patients with type 2 diabetes mellitus (T2DM) simultaneously suffer coronary artery disease (CAD) [1]. Patients with CAD are at significantly higher risk of adverse events once combined with diabetes mellitus (DM). The accurate identification and subsequent treatment of high-risk patients with CAD and DM to reduce adverse prognosis has been a hotspot of current research. Inflammation plays a crucial role in the progression of coronary atherosclerotic disease and DM. There is growing evidence that the higher levels of inflammatory biomarkers are significantly associated with an increased risk of adverse cardiovascular events in patients with CAD or DM [2–4].

As acute phase proteins of inflammation, both high-sensitivity C-reactive protein (hs-CRP) and albumin are vital indicators that reflect the inflammation grade. However, their acute phase responses to inflammation are distinct, with hs-CRP levels rising and albumin levels falling [5]. Previous studies have explored hs-CRP and albumin and prognosis of CAD, respectively. On the one hand, in patients with CAD, it was shown that hs-CRP was strongly associated with disease severity [6] and adverse cardiovascular events [2, 3]. On the other hand, in patients with T2DM, hs-CRP was associated with an increased risk of adverse cardiovascular events [7] and mortality [8]. Albumin is an important nutritional indicator that is associated with inflammatory and hemostatic processes. Hypoalbuminemia is an independent risk factor for in-hospital and long-term prognosis in patients with acute coronary syndromes (ACS) and myocardial infarction (MI) [9–11]. Therefore, early prediction of the effect of inflammation on the risk of death and cardiovascular events in PCI patients combined with T2DM (individuals at high risk among patients with CAD [12]) is an essential clinical issue. The hs-CRP-to-albumin ratio (CAR) is a very novel indicator of inflammation, but to date, the association between CAR levels and long-term prognosis in a diabetic population with PCI has not been reported in the literature.

Recent studies have shown that CAR is a more accurate indicator than albumin and hs-CRP alone in terms of the evaluation of systemic inflammatory status as well as the determination of prognosis of patients with cancer and critical illness. CAR is a prognostic factor in esophageal [13], hepatocellular [14], and ovarian cancers [15]. In patients with CAD, it is strongly associated with the disease severity [16, 17], coronary thrombotic load [18] and prognosis [19–21]. It is an important clinical issue to assess whether CAR can predict the risk of all-cause mortality and cardiac mortality in PCI patients with T2DM. Therefore, we aimed to use a large, real-world and long-term follow-up dataset to evaluate the effect of CAR on the risk of long-term adverse cardiovascular events in patients with T2DM treated with PCI.

## Methods

### Study design and patients

**Fig. 1:**
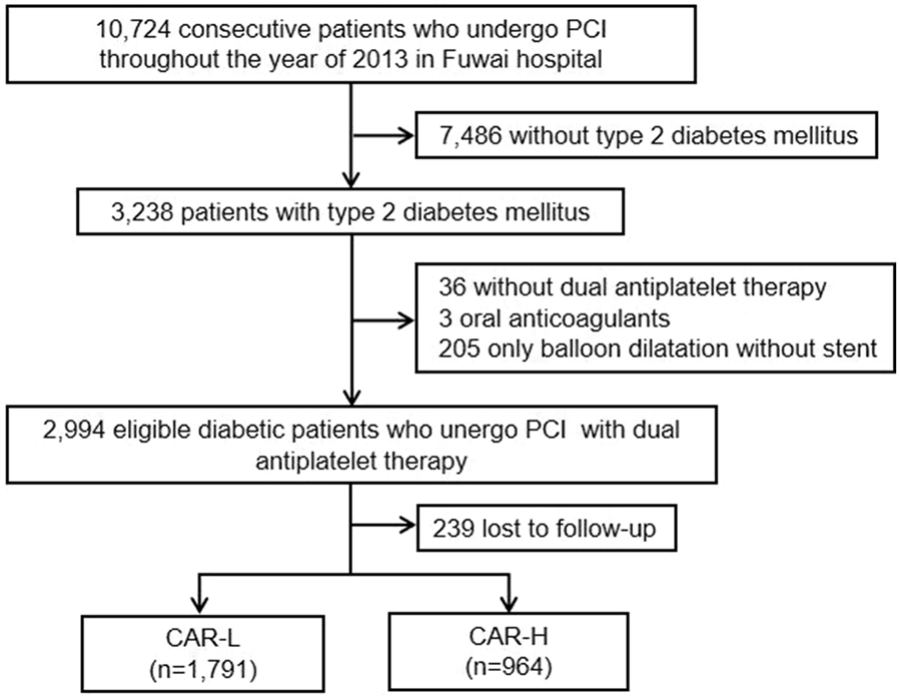
A flow chart of the study. PCI: percutaneous coronary intervention, DAPT: dual antiplatelet therapy, CAR: hs-CRP to albumin ratio

The present study was based on a real-world, prospective, single-centre and observational cohort. From January 2013 to December 2013, 10,724 patients who were treated with PCI were consecutively and prospectively enrolled in Fu Wai Hospital, National Center for Cardiovascular Diseases, National Clinical Research Center for Cardiovascular Diseases and State Key Laboratory of Cardiovascular Diseases (Beijing, China). Among them, 3238 patients with T2DM. After excluding 36 patients not treated with dual antiplatelet therapy (DAPT) at baseline, 3 patients treated with oral anticoagulants and 205 who received only balloon dilatation without stent, 2994 eligible diabetic patients who undergo PCI with DAPT were enrolled. Furthermore, we excluded 239 patients lost to follow-up, with a final sample size of 2755 (92.0%) for statistical analysis (Fig. 1).

After PCI, DAPT consisting of aspirin 100 mg daily and clopidogrel 75 mg daily or ticagrelor 90 mg twice daily were administered for at least 12 months in all participants according to the guideline at that time in China [22]. This study complied with the Helsinki Declaration. The Review Board of Fu Wai Hospital approved the study protocol (Approval Number: 2013-449). All participants provided written informed consent.

### Parameters measurements

Within 24 h after admission, venous blood was collected from all patients after fasting and was sent to the laboratory within two hours. The serum albumin concentration was measured by an AU5400 automatic biochemical analyzer (Beckman Coulter, Brea, CA, USA), and the serum hs-CRP concentration was measured by an automated biochemical analyzer (Labospect 008, Hitachi 7150, Tokyo, Japan) in the Clinical Chemistry Department of Fu Wai Hospital.

### Definition and study outcomes

CAR refers to the hs-CRP to albumin ratio. According to the optimal cut-off value of CAR for all-cause mortality, patients with higher CAR were divided into a higher level of CAR (CAR-H) group, while those with lower CAR were divided into a lower level of CAR (CAR-L) group.

The primary endpoint was all-cause mortality. The secondary endpoint was cardiac mortality. Follow-ups were regularly conducted through clinic visits or by telephone interviews at 30 days, 6 months, 1 year, 2 years and 5 years, with a 92.0% follow-up rate at 5 years. The time to events was calculated as the number of days from the date of performing PCI to the date of the death, the last visit or the last recorded clinical event of participants still alive, whichever came first. All endpoint events were adjudicated centrally by two independent cardiologists, and possible disagreement was resolved by consensus.

### Statistical analysis

Continuous variables with a normal distribution were expressed as mean ± standard deviation, while categorical variables were expressed as frequency (percentage). Continuous variables were compared by the Student’s t-test, and the Pearson chi-square test or Fisher’s exact test was used to compare categorical variables.

Receiver operating characteristic (ROC) curve analysis was performed to obtain the area under the curve (AUC) and the Youden’s index. The Youden’s index with the greatest sensitivity and specificity was used to determine the optimal cut-off value for CAR to predict long-term all-cause mortality. Survival curves were constructed by Kaplan–Meier method and compared by the log-rank test. The univariate and multivariate Cox regression analyses were conducted to calculate the Hazard ratios (HRs) and 95% confidence interval (CI) and assess the associations among CAR and 5-year outcomes. The multivariate Cox model was adjusted for the following covariates: sex, body mass index, hypertension, ACS, haemoglobin, white blood cell count, estimated glomerular filtration rate and glucose. Furthermore, to reduce confounding, propensity score matching was performed to adjust for baseline differences between CAR-L and CAR-H groups. The exploratory analyses were performed to assess the effect of CAR in different subgroups on the primary endpoint. Pearson correlation and linear regression analysis were constructed to evaluate the correlation between hs-CRP and albumin. The prediction of CAR and hs-CRP for 5-year outcomes were assessed by using AUC and compared by the Delong test. All statistical analyses were performed at a significance level of two-sided 0.05. Statistical analyses were performed via SPSS 23.0 (IBM Corp., Armonk, New York, USA), R Programming Language version 4.0.3 (R Core Team, 2014) and GraphPad Prism version 8.0.0 for windows (GraphPad Software, San Diego, California USA).

## Results

A total of 2755 eligible diabetic patients who undergo PCI with DAPT were included for final analysis (Fig. 1), among whom 126 (4.6%) experienced all-cause mortality and 74 (2.7%) experienced cardiac mortality during a follow-up of 5 years.

### Baseline characteristics

The mean age of the 2755 participants was 59.2 ± 9.7 years, 2060 (74.8%) were men and 1552 (56.3%) clinically presented with ACS. Based on the Youden’s index, the optimal cut-off point of CAR is 0.0594. Table 1 shows the comparison of baseline characteristics of CAR-H versus CAR-L. Individuals with higher CAR had less men, a significantly higher body mass index, a higher prevalence of hypertension and ACS, a lower baseline haemoglobin, a higher baseline white blood cell count, a lower baseline estimated glomerular filtration rate and a higher baseline glucose concentration than those with lower CAR (all p < 0.05).

**Table 1:** Baseline characteristic

Variables	CAR-H (n=964)	CAR-L (n=1791)	P Value
Demographics			
Age (years)	59.6±9.8	59.0±9.6	0.100
Men	690 (71.6)	1370 (76.5)	0.005
Body mass index (kg/m^2^)	26.7±3.2	26.2±3.1	<0.001
Cardiovascular risk factor			
Previous PCI	242 (25.1)	495 (27.6)	0.152
Previous CABG	55 (5.7)	76 (4.2)	0.085
Hypertension	706 (73.2)	1218 (68.0)	0.004
COPD	26 (2.7)	36 (2.0)	0.246
PVD	34 (3.5)	72 (4.0)	0.521
Previous stroke	143 (14.8)	223 (12.5)	0.079
Previous MI	174 (18.0)	377 (21.0)	0.060
Current/ever-smoker	560 (58.1)	984 (54.9)	0.112
Initial presentation			
Clinical presentation			<0.001
ACS	613 (63.6)	939 (52.4)	
CCS	351 (36.4)	852 (47.6)	
Laboratory results at admission			
Haemoglobin (g/dL)	14.0±1.7	14.3±1.5	<0.001
White blood cell count (10^9^/L)	7.7±2.2	6.6±1.6	<0.001
eGFR (ml/min)	88.9±17.2	91.7±15.1	<0.001
Glucose (mmol/L)	8.2±2.9	7.5±2.5	<0.001
Procedural presentation			
SYNTAX score^a^	12.4±8.3	12.1±8.3	0.393
GPI	144 (14.9)	295 (16.5)	0.294
Medication at discharge			
Clopidogrel	964 (100.0)	1786 (99.7)	0.170
Statin	927 (96.2)	1708 (95.4)	0.329
Beta-blocker	880 (91.3)	1646 (91.9)	0.575

CAR: hs-CRP to albumin ratio, PCI: percutaneous coronary intervention, CABG: coronary artery bypass grafting, COPD: chronic obstructive pulmonary disease, PVD: peripheral vascular disease, MI: myocardial infarction, ACS: acute coronary syndrome, CCS: chronic coronary syndrome, eGFR: estimated glomerular filtration rate, SYNTAX: (synergy between percutaneous coronary intervention with taxus and cardiac surgery, GPI: glycoprotein IIb/IIIa inhibitor

^a^Calculated using an online calculator (http://www.syntaxscore.com) by a dedicated research group blinded to the clinical data

### Incidences of 5-year outcomes in CAR-L and CAR-H

The incidences of all-cause mortality (62/964 [6.4%] versus 64/1791 [3.6%], p = 0.001) and cardiac mortality (39/964 [4.0%] versus 35/1791 [2.0%], p = 0.001) were significantly higher in patients with CAR-H than in those with CAR-L.

### Survival analysis

**Fig. 2:**
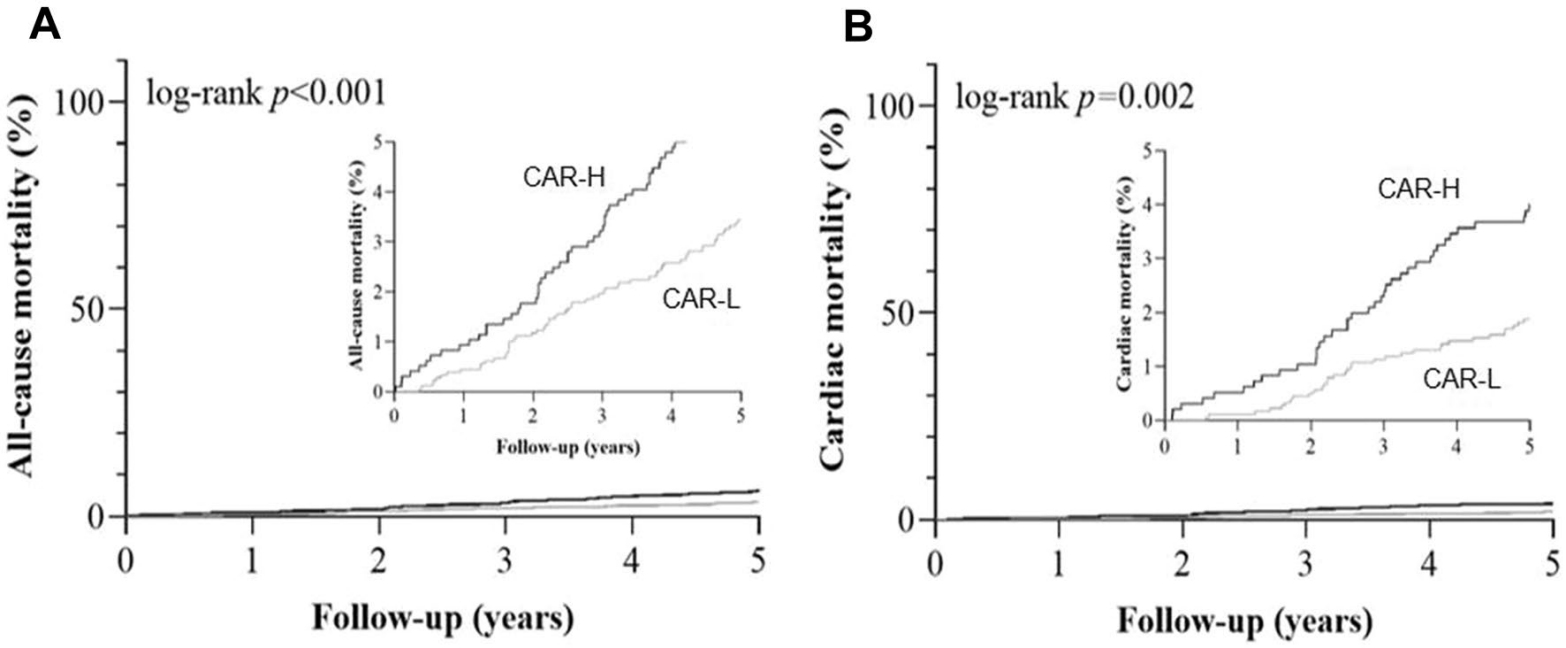
Kaplan–Meier estimates of the cumulative incidences for 5-year outcomes by strata of CAR. (**A–B**) Kaplan–Meier estimates of the cumulative incidences for all-cause mortality (**A**) and cardiac mortality (**B**). CAR: hs-CRP to albumin ratio

The Kaplan–Meier estimates of all-cause mortality and cardiac mortality were shown in Fig. 2. In the univariate Cox model, CAR-H was associated with all-cause mortality (HR: 1.815, 95% CI 1.280–2.574, p < 0.001) and cardiac mortality (HR: 2.085, 95% CI 1.321–3.291, p = 0.002) compared with CAR-L (Table 2). In the multivariable Cox model, patients with higher CAR had a higher risk of all-cause mortality (adjusted HR: 1.634, 95% CI 1.121–2.380, p = 0.011) and a higher risk of cardiac mortality (adjusted HR: 1.733, 95% CI 1.059–2.835, p = 0.029) compared with those with lower CAR (Table 3).

**Table 2:** Univariate analysis of the association between CAR-H and 5-year outcomes

Covariates	All-cause mortality		Cardiac mortality	
	HR (95% CI)	P value	HR (95% CI)	P value
CAR-H	1.815 (1.280–2.574)	<0.001	2.085 (1.321–3.291)	0.002
Age	1.060 (1.041–1.080)	<0.001	1.058 (1.032–1.084)	<0.001
Men	1.187 (0.780–1.806)	0.424	1.231 (0.708–2.141)	0.461
Body mass index	1.005 (0.951–1.063)	0.848	1.039 (0.967–1.116)	0.295
Previous PCI	1.599 (1.113–2.298)	0.011	1.892 (1.190–3.010)	0.007
Previous CABG	0.990 (0.436–2.248)	0.981	0.833 (0.263–2.646)	0.757
Hypertension	1.530 (1.005–2.328)	0.047	1.583 (0.910–2.753)	0.104
COPD	4.426 (2.384–8.216)	<0.001	5.581 (2.679–11.630)	<0.001
PVD	1.464 (0.683–3.139)	0.327	1.428 (0.521–3.911)	0.488
Previous Stroke	1.478 (0.940–2.322)	0.090	1.411 (0.775–2.568)	0.260
Previous MI	1.619 (1.100–2.383)	0.015	2.465 (1.541–3.944)	<0.001
Current/ever-smoker	1.279 (0.893–1.833)	0.180	1.744 (1.066–2.854)	0.027
ACS	0.830 (0.585–1.177)	0.296	1.020 (0.644–1.616)	0.932
Haemoglobin	0.985 (0.974–0.996)	0.008	0.979 (0.965–0.993)	0.004
White blood cell count	1.062 (0.977–1.156)	0.159	1.074 (0.964–1.196)	0.198
eGFR	0.970 (0.961–0.979)	<0.001	0.962 (0.951–0.972)	<0.001
Glucose	1.047 (0.985–1.112)	0.140	1.056 (0.978–1.141)	0.164
SYNTAX Score	1.000 (0.980–1.022)	0.968	0.995 (0.967–1.023)	0.705
GPI	0.870 (0.528–1.432)	0.584	0.723 (0.360–1.451)	0.361
Clopidogrel	NA	NA	NA	NA
Statin	1.102 (0.451–2.696)	0.831	1.642 (0.403–6.691)	0.489
Beta-blocker	0.856 (0.472–1.552)	0.609	0.652 (0.325–1.309)	0.229

HR: hazard ratio, CI: confidence interval, NA: not applicable. Meanings of other abbreviations are identical to those in Table 1

**Table 3:** Multivariate analysis of the association between CAR-H and 5-year outcomes

5-year outcomes	All-cause mortality		Cardiac mortality	
	HR (95% CI)	P value	HR (95% CI)	P value
CAR-L	Reference	NA	Reference	NA
CAR-H	1.634 (1.121–2.380)	0.011	1.733 (1.059–2.835)	0.029

The multivariate Cox model was adjusted for the following covariates: sex, body mass index, hypertension, ACS, haemoglobin, white blood cell count, estimated glomerular filtration rate and glucose

HR: hazard ratio, CI: confidence interval, NA: not applicable

### ROC curves of CAR to 5-year follow-up

**Fig. 3:**
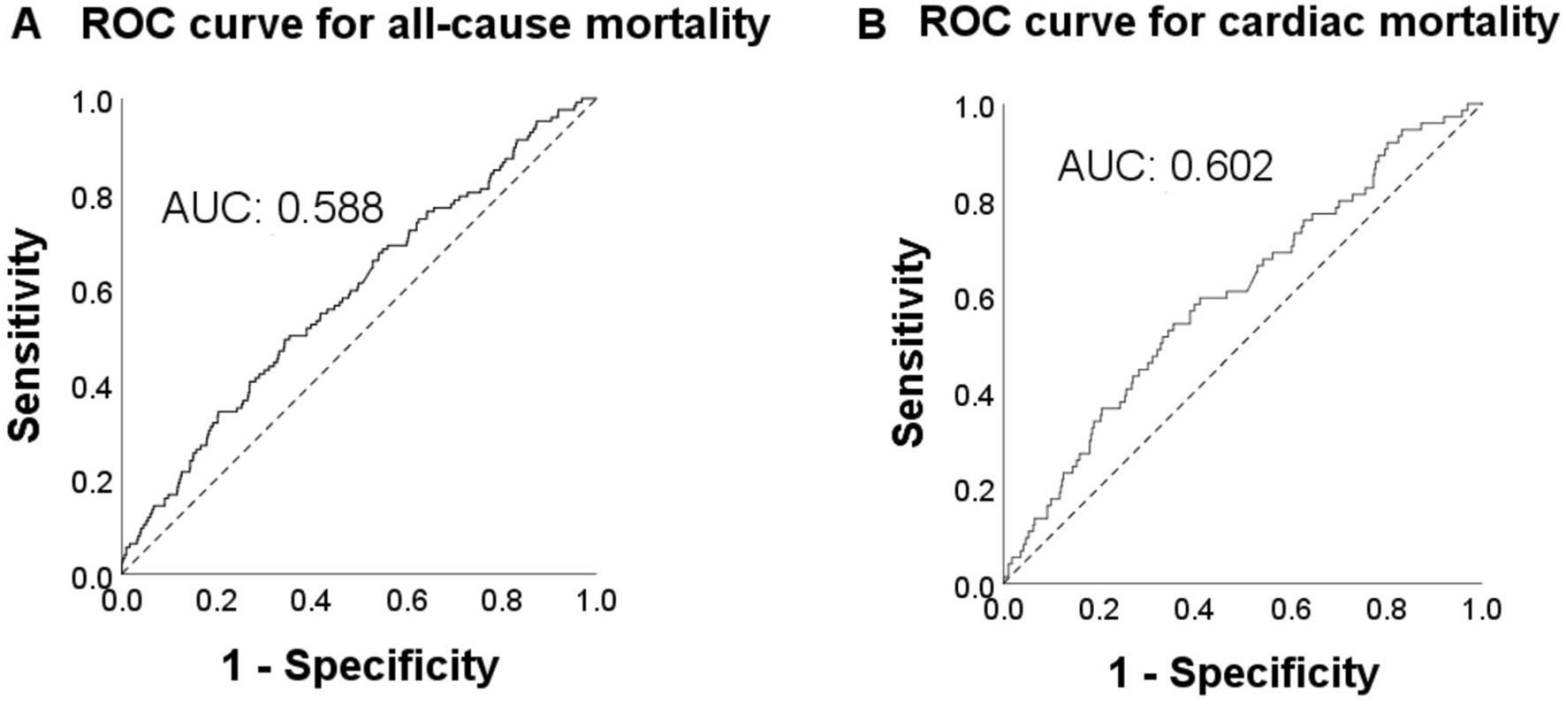
ROC curves of CAR for 5-year outcomes. (**A**–**B**) ROC curves of CAR for 5-year all-cause mortality (**A**); and cardiac mortality (**B**). ROC: receiver operating characteristic, AUC: area under the curve

ROC curves were performed to assess the predictive value of CAR for 5-year outcomes as a continuous variable. The CAR showed a predictive value for all-cause mortality with an AUC of 0.588 (95% CI 0.536–0.639), and for cardiac mortality with an AUC of 0.602 (95% CI 0.537–0.667) (Fig. 3).

### The comparison between CAR and hs-CRP

The prediction of CAR for all-cause mortality was superior to hs-CRP (AUC 0.588 vs. 0.580, Z=− 3.140, p = 0.002) and the prediction of CAR for cardiac mortality was superior to hs-CRP (AUC 0.602 vs. 0.593, Z=− 2.889, p = 0.004).

### Sensitivity analysis

A 1:2 propensity score matching cohort (n = 2122) was obtained from CAR-H (n = 964) versus CAR-L (n = 1791). This analysis showed CAR-H was associated with all-cause mortality (HR:1.484, 95% CI 1.000–2.201) and cardiac mortality (HR: 1.691, 95% CI 1.001–2.856) compared with CAR-L (Additional file 1: Table S1).

### Subgroup analyses

**Fig. 4:**
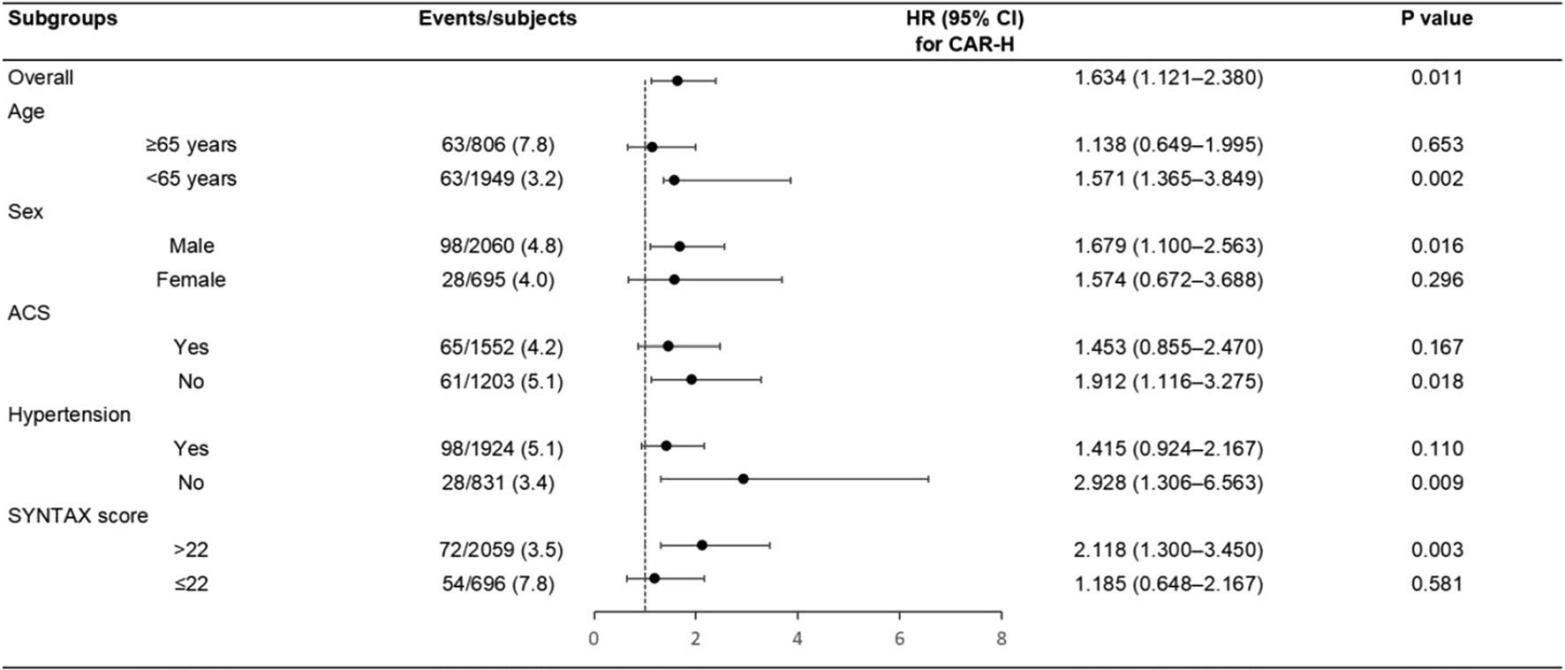
Subgroup analyses. ACS: acute coronary syndrome, SYNTAX: synergy between percutaneous coronary intervention with taxus and cardiac surgery, HR: hazard ratio, CI: confidence interval, CAR: hs-CRP to albumin ratio

Subgroup analyses showed the CAR-H was associated with all-cause mortality relative to CAR-L in certain subsets (age, sex, ACS, hypertension, and SYNTAX score) (Fig. 4).

### Correlation analysis between hs-CRP and albumin

The range for albumin was 24.20 to 55.30 g/L. Only 4 (0.1%) patients showed hypoalbuminemia (albumin < 30 g/L). The range for hs-CRP was 0–16.99 mg/L. No patient with hs-CRP > 20 mg/L. Pearson correlation analysis and linear regression analysis showed that hs-CRP was negatively associated with albumin (R^2^ = 0.043, Standard β=− 0.208, p < 0.001) (Additional file 1: Figure S1).

## Discussion

This real-world study of 2755 participants with T2DM who undergo PCI with DAPT with 5 years of follow-up showed that compared with patients with a lower level of CAR, those with a higher level of CAR was associated with an increased risk of long-term mortality including all-cause mortality and cardiac mortality; the prediction of CAR for all-cause mortality and cardiac mortality was superior to hs-CRP; the results remained consistent after propensity score matching.

### CAR is associated with the risk of long-term mortality in patients with CAD combined with T2DM

CAR is a novel biomarker of inflammation. The present study is the first to report that although receiving PCI and DAPT, CAR still can reflect long-term residual inflammation risk in patients with CAD combined with T2DM. CAR is composed of two inflammatory factors: hs-CRP and albumin. In recent years, the association of hs-CRP and albumin and poor prognosis in patients with CAD has been a hotspot, yielding relatively consistent findings. Many previous studies have shown that hs-CRP is strongly associated with a higher incidence of long-term ischemic events in patients with CAD treated with PCI [2, 3], and decreased serum albumin is associated with susceptibility and poor prognosis of cardiovascular disease [9–11, 23]. Wada et al. showed that the presence of low serum albumin and high CRP levels had a synergistic effect on elevated long-term ischemic risk in patients undergoing PCI [24]. Meanwhile, in studies of diabetic patients, a recent meta-analysis has shown that hs-CRP is associated with a high risk of developing T2DM [4]. It has also been shown that chronic low-grade inflammation, as determined by measurement of hs-CRP, plays an important role in the development of T2DM [25] and may be a therapeutic target for reducing residual cardiovascular risk in patients with T2DM [26]. Albumin is an important biomarker for monitoring the pathophysiology of DM [27]. The albumin levels are reduced by approximately 20% in diabetic patients compared to normal subjects and basic trials have shown that treatment of diabetic rats with insulin restores albumin to normal levels [28].

CAR is a significant inflammatory biomarker for assessing prognosis in patients with certain cancer [15, 29–31], and has been shown to have a better predictive value than hs-CRP and albumin alone in terms of ischemic events [21, 32]. Meanwhile, CAR has a better diagnostic value for CAD than other inflammatory indicators (neutrophil to lymphocyte ratio, and monocyte to lymphocyte ratio, etc.) [33] and can be used as a predictor of atherosclerosis and CAD [34]. To date, there have been several studies on the prognostic value of CAR in CAD. However, it is worth noting that firstly, the number of people with CAD included in these studies was small, with the majority of the study population being only a few hundred, except for three which included 1396 [19], 1630 [20] and 2243 [21] patients. Secondly, the duration of follow-up was short. The investigators observed mostly in-hospital or short-term events with the longest mean follow-up being 37.59 months [20]. Finally, none of these studies performed further subgroup analyses in the DM population, so the predictive value of CAR in patients with CAD combined with DM is unclear. In terms of death, Liu et al. [20] showed that CAR was an independent predictor of long-term all-cause mortality and cardiac death in patients with CAD undergoing PCI (n = 1630). Among patients with ST-segment elevated MI (STEMI), Söğüt et al. (n = 116) [35] and Çınar et al. (n = 2,243) [21] showed that CAR was an independent predictor of in-hospital mortality and long-term all-cause mortality, respectively. Cheng et al. [36] found in patients with coronary chronic total occlusion (n = 664) that patients with higher CAR had an increased risk of all-cause mortality. In addition to the ischaemic aspect of death. Acet et al. [32] showed that CAR was an independent predictor of major adverse cardiac events (MACE) at 6 months in patients with STEMI (n = 539). Wang et al. [37] showed that CAR was independently associated with in-hospital and short-term MACE in patients with ACS (n = 652). Aksu et al. [19] found in STEMI patients (n = 1396) that CAR was an independent predictor of stent restenosis.

All of the above studies support the predictive value of CAR for ischaemic adverse events in patients with CAD. It has been suggested that CAR is a predictor of diabetes mellitus [38] and that higher CAR significantly increases the risk of the serious postoperative complication “systemic inflammatory response syndrome”, which is further increased in elderly patients with DM [39]. In summary, it is important to explore the effect of CAR on long-term prognosis in PCI patients with CAD combined with DM. However, there is a lack of literature on this subject. Therefore, in this study, 2755 patients with T2DM were selected from 10,724 patients with CAD treated with PCI for analysis. Our results showed that higher levels of CAR were strongly associated with a higher risk of long-term all-cause mortality and cardiac mortality in diabetic patients with PCI and that CAR was independently associated with poor prognosis, regardless of ACS.

Compared to previous CAR-related studies in patients with CAD, the total population with CAD enrolled in this study (n = 10,724) is the largest in number in this field to date and is more representative. It is worth pointing out that this study focused on patients at higher risk in the population with CAD (T2DM). Besides, this result remained consistent after propensity score matching, indicating reliable stability. Patients with CAD combined with diabetes represent a larger proportion of the population, and assessing CAR in this group of patients may have clinical implications for the management of high-risk individuals. Diabetics account for a large proportion of patients with CAD [12], and the assessment of CAR in these patients may be of clinical implications for the management of high-risk individuals.

### Potential mechanisms and clinical implications

Potential mechanisms to the pathophysiology of elevated CAR associated with poor prognosis of death in patients with PCI combined with T2DM are as follows. Our previous studies have shown that higher levels of hs-CRP are associated with high platelet reactivity [40], which is associated with an increased risk of ischaemic events [41]. In addition, CRP promotes smooth muscle cell proliferation, affects human macrophage polarization [42] enhances thromboxane activity [42] and ultimately leads to atherosclerosis and thrombosis. Albumin has a number of important physiological functions, such as antioxidant and anticoagulant functions [43]. With regard to the characteristic of antioxidants, serum albumin contains a free cysteine at the − 34 position, called the Cys34 residue, which has redox properties [44]. With this, serum albumin inhibits lipid peroxidation [45] and increases glutathione levels [46], acting as an anti-atherogenic agent. With regard to the characteristic of anticoagulation, albumin Cys-34 binds nitric oxide, resulting in the formation of nitrosoalbumin, which prolongs the biological activity of nitric oxide, acting as a vasodilator and inhibitor of platelet aggregation agent [47]. In particular, serum albumin in diabetic patients is glycated, impairing its ligand to bind to albumin and further impairing antioxidant and anticoagulant properties. Although we know that inflammatory hypotheses have been proposed in the development of CAD and DM, the exact mechanisms linking high hs-CRP and low serum albumin to increased risk of CAD and DM are not yet clear. Therefore, further studies are needed to elucidate the underlying mechanisms.

In conclusion, this study showed that patients with T2DM treated with PCI who have higher CAR levels were associated with a higher risk of long-term mortality (both all-cause and cardiac mortality) compared to those with lower CAR levels. In the future, it’s necessary to routinely screen this simple and readily available novel inflammatory biomarker, CAR, in patients with DM after PCI. Controlling residual inflammation risk can have further cardiovascular benefits, and anti-inflammatory therapy has yielded some encouraging results in recent years [48], but to our knowledge, anti-inflammatory drugs mostly focus on the levels of hs-CRP and interleukin-1β. Our research points out that in the future, we might pay attention to serum albumin levels at the same time, especially in patients with type 2 diabetes who undergo PCI. In other words, we are looking forward to a therapeutic target aimed at simultaneously lowering hs-CRP levels and raising serum albumin levels. In the future, whether long-term control of CAR levels can reduce the risk of events in patients with CAD combined with DM is worth further study.

### Limitations

Our study had several limitations. First, this was a single-centre, observational study. Therefore, this study has the inherent defects of an observational study, and the extrapolation of our conclusions requires further verification. Second, we did not routinely evaluate serum hs-CRP and albumin concentrations which will fluctuate normally during the follow-up. Third, although we attempted to adjust as many important confounding factors as possible, unmeasured confounding factors still cannot be ruled out as related to the risk of outcomes. Fourth, the discrimination of CAR for all-cause mortality and cardiac mortality was poor to fair.

## Conclusion

This large-sample, real-world study shows that higher level of CAR was associated with the risk of 5-year all-cause mortality and cardiac mortality in diabetic patients who undergo PCI with DAPT. CAR showed a predictive value for 5-year outcomes, which was superior to hs-CRP. This finding may shed a light on better management of patients with PCI, indicating that more attention should be paid to the inflammatory marker CAR in treatment in the current era of DAPT.

## Supplementary information

**Additional file 1: Figure S1.** Correlation analysis between hs-CRP and albumin. **Table S1.** Propensity score-matched analysis.

## Data Availability

Due to ethical restrictions related to the consent given by subjects at the time of study commencement, our datasets are available from the corresponding author upon reasonable request after permission of the Institutional Review Board of State Key Laboratory of Cardiovascular Disease, Fuwai Hospital, National Center for Cardiovascular Diseases.

## Abbreviations

ACS: Acute coronary syndrome
DAPT: Dual antiplatelet therapy
DM: Diabetes mellitus
T2DM: Type 2 diabetes mellitus
CAD: Coronary artery disease
hs-CRP: High-sensitivity C reactive protein
PCI: Percutaneous coronary intervention
MI: Myocardial infarction
STEMI: ST-segment elevated myocardial infarction
CAR: hs-CRP-to-albumin ratio
ROC: Receiver operating characteristic
AUC: Area under the curve
HR: Hazard ratio
CI: Confidence interval
MACE: Major adverse cardiac events
