# Supplementary material for: A novel inflammatory biomarker, high-sensitivity C-reactive protein-to-albumin ratio, is associated with 5-year outcomes in patients with type 2 diabetes who undergo percutaneous coronary intervention
Source: Diabetol Metab Syndr. 2023 Feb 6;15:14. doi: 10.1186/s13098-022-00977-9 (PMC9901126; doi:10.1186/s13098-022-00977-9)
Supplement: Supplementary file 1 — Additional file 1: Figure S1. Correlation analysis between hs-CRP and albumin. Table S1. Propensity score-matched analysis. [file 13098_2022_977_MOESM1_ESM.docx]

**Additional file 1**

Li et al. A novel inflammatory biomarker, high-sensitive C-reactive protein-to-albumin ratio, is associated with 5-year outcomes in patients with type 2 diabetes who undergo percutaneous coronary intervention

**Contents**

**Figure S1.** Correlation analysis between hs-CRP and albumin

**Table S1.** Propensity score-matched analysis

**Figure S1.** Correlation analysis between hs-CRP and albumin


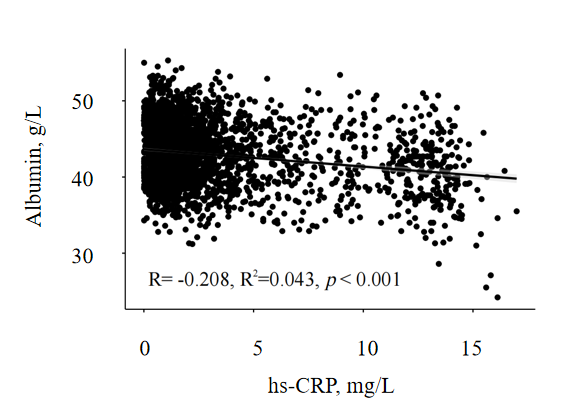


hs-CRP: high-sensitivity C reactive protein.

**Table S1.** Propensity score-matched analysis

| Outcomes | Events/subjects | HR (95% CI) | P Value |
| --- | --- | --- | --- |
| All-cause mortality | 99 (4.7) | 1.484 (1.000–2.201) | <0.050 |
| Cardiac mortality | 56 (2.6) | 1.691 (1.001–2.856) | <0.050 |

HR: hazard ratio; CI: confidence interval.
